# Case Report: Circulating Myeloid-Derived Suppressive-Like Cells and Exhausted Immune Cells in Non-Small Cell Lung Cancer Patients Treated With Three Immune Checkpoint Inhibitors

**DOI:** 10.3389/fimmu.2021.672219

**Published:** 2021-05-27

**Authors:** Giuseppe Bronte, Alberto Verlicchi, Serena De Matteis, Alice Rossi, Alessandra Affatato, Francesco Giulio Sullo, Caterina Gianni, Matteo Canale, Marco Angelo Burgio, Angelo Delmonte, Michele Milella, Lucio Crinò

**Affiliations:** ^1^ Department of Medical Oncology, IRCCS Istituto Romagnolo per lo Studio dei Tumori (IRST) “Dino Amadori”, Meldola, Italy; ^2^ Biosciences Laboratory, IRCCS Istituto Romagnolo per lo Studio dei Tumori (IRST) “Dino Amadori”, Meldola, Italy; ^3^ Department of Experimental, Diagnostic and Specialty Medicine, AlmaMater Studiorum, University of Bologna, Bologna, Italy; ^4^ Radiology Unit, IRCCS Istituto Romagnolo per lo Studio dei Tumori (IRST) “Dino Amadori”, Meldola, Italy; ^5^ Unit of Biostatistics and Clinical Trials, IRCCS Istituto Romagnolo per lo Studio dei Tumori (IRST) “Dino Amadori”, Meldola, Italy; ^6^ Section of Oncology, Department of Medicine, University of Verona, Verona, Italy

**Keywords:** immune checkpoint inhibitor, CTLA-4, PD-1, LAG-3, myeloid-derived suppressor cells, T cell exhaustion

## Abstract

Immune checkpoint inhibition induced a great step forward in the treatment of non-small cell lung cancer patients. In cancer immune microenvironment many checkpoints were studied and their involvement could represent a mechanism of resistance to cancer immunotherapy. For this reason, the inhibition of multiple immune checkpoints is under development. However, myeloid-derived suppressor cells (MDSC) and exhausted immune cells could limit the efficacy of cancer immunotherapy. We analyzed the variation of circulating immune suppressive-like cell subsets and exhausted immune cells in three non-small cell lung cancer patients treated with the combination of anti-CTLA-4 plus anti-PD-1 plus anti-LAG-3 at T0 (baseline), T1 (after 2 months) and T2 (after 4 months). We also describe the clinical and radiological course of the disease during this treatment in all three patients. We observed both clinical differences and changes in the composition of immune suppressive-like cell subsets and exhausted immune cells between the patients receiving the same schedule of treatment with immune checkpoint inhibitors. The study on a wider patient population and experimental model design could help to clarify the kinetics of these cell subpopulations with the perspective to find new targets for treatment or new biomarkers for resistance to cancer immunotherapy.

## Introduction

The discovery of immune checkpoints, as a mechanism of immune escape in cancer immunology, allowed to develop a new treatment strategy with specific inhibitors of these molecules. Some immune checkpoints are the Cytotoxic T-Lymphocyte Antigen 4 (CTLA-4), the programmed death 1 (PD-1)/PD-1 ligand (PD-L1) axis and the Lymphocyte-activation gene 3 (LAG-3). Currently, the immune checkpoint inhibitors (ICIs) which are approved for patients with advanced non-small cell lung cancer (NSCLC) include the PD-1 inhibitors pembrolizumab, nivolumab and the PD-L1 inhibitor atezolizumab and durvalumab (1).

The treatment for advanced NSCLC is now based on the association of an ICI with platinum-based chemotherapy, given that the combination of pembrolizumab with pemetrexed and cisplatin or carboplatin has already been approved for clinical practice in patients with non-oncogene-addicted advanced NSCLC. The Food and Drug Administration (FDA) approved the combination of nivolumab plus ipilimumab as first-line treatment for patients with metastatic NSCLC and PD-L1 ≥ 1% expression (2).

PD-L1 expression in cancer cells, as determined by immunohistochemistry is currently the only approved predictive biomarker for anti-PD-1 therapy between patients who would or would not benefit from anti-PD-1 therapy. However, less than one half of the patients selected because of a high PD-L1 expression respond to a ICI as well as many patients with negative PD-L1 expression experienced a response (3). Many other biomarkers are under investigation. Among these, the tumor mutational burden (TMB) achieved more interest than others. In the phase III trial CheckMate 227, the combination of the anti-CTLA-4 ipilimumab and the anti-PD-1 nivolumab achieved better outcomes in comparison with chemotherapy in the subgroup of patients with a high TMB (≥ 10 mutations/Mb) (4).

Considering the low accessibility of tumor tissues from patients and tumor heterogeneity depending on the biopsy site, the research for new predictive biomarkers in peripheral blood would be ideal for clinical application as a non-invasive and simple method.

To date, we need a deeper knowledge about the underlying mechanisms of immune escape and therapeutic resistance to ICIs that occurs in the majority of patients, leading to tumor progression (5, 6). One important resistance mechanism is installed by several immunosuppressive factors and cells, including myeloid-derived suppressor cells (MDSC) and T cell exhaustion (7, 8).

MDSCs are considered an obstacle for many cancer immunotherapies. Consequently, numerous studies are focused on the characterization of MDSC origin and their relationship to other myeloid cell populations, their immunosuppressive capacity, and possible ways to inhibit MDSC function with different approaches being evaluated in clinical trials (9).

It has been reported that the frequency of circulating regulatory T cells and MDSC correlated with response to anti-PD-1 therapy in NSCLC patients (9).

During chronic infections and cancer characterized by a persistent antigen exposure and/or inflammation, an altered differentiation state, termed T cell exhaustion manifests with several features, such as loss of effector functions, sustained upregulation and co-expression of multiple inhibitory receptors.

Here, we report three clinical cases of advanced NSCLC patients receiving a combined treatment with anti-CTLA-4 ipilimumab, anti-PD-1 nivolumab and anti-LAG-3 relatlimab. Together with the description of clinical presentation, we depict circulating MDSC and exhausted T, NK and NKT cells over the course of treatment.

## Medical History and Diagnostics

### Clinical Presentation of Case 1

A 70-years-old male patient was diagnosed in May 2019 with advanced lung adenocarcinoma. The baseline Computed Tomography (CT) scan showed bilateral lung lesions and the involvement of mediastinal lymph nodes (T0). Bronchoscopy was performed leading to the diagnosis of poorly differentiated adenocarcinoma. The molecular profile was negative for EGFR mutation, ALK and ROS1 rearrangements and PD-L1 expression. A treatment with the immunological triplet was started in June 2019.

**Figure 1:**
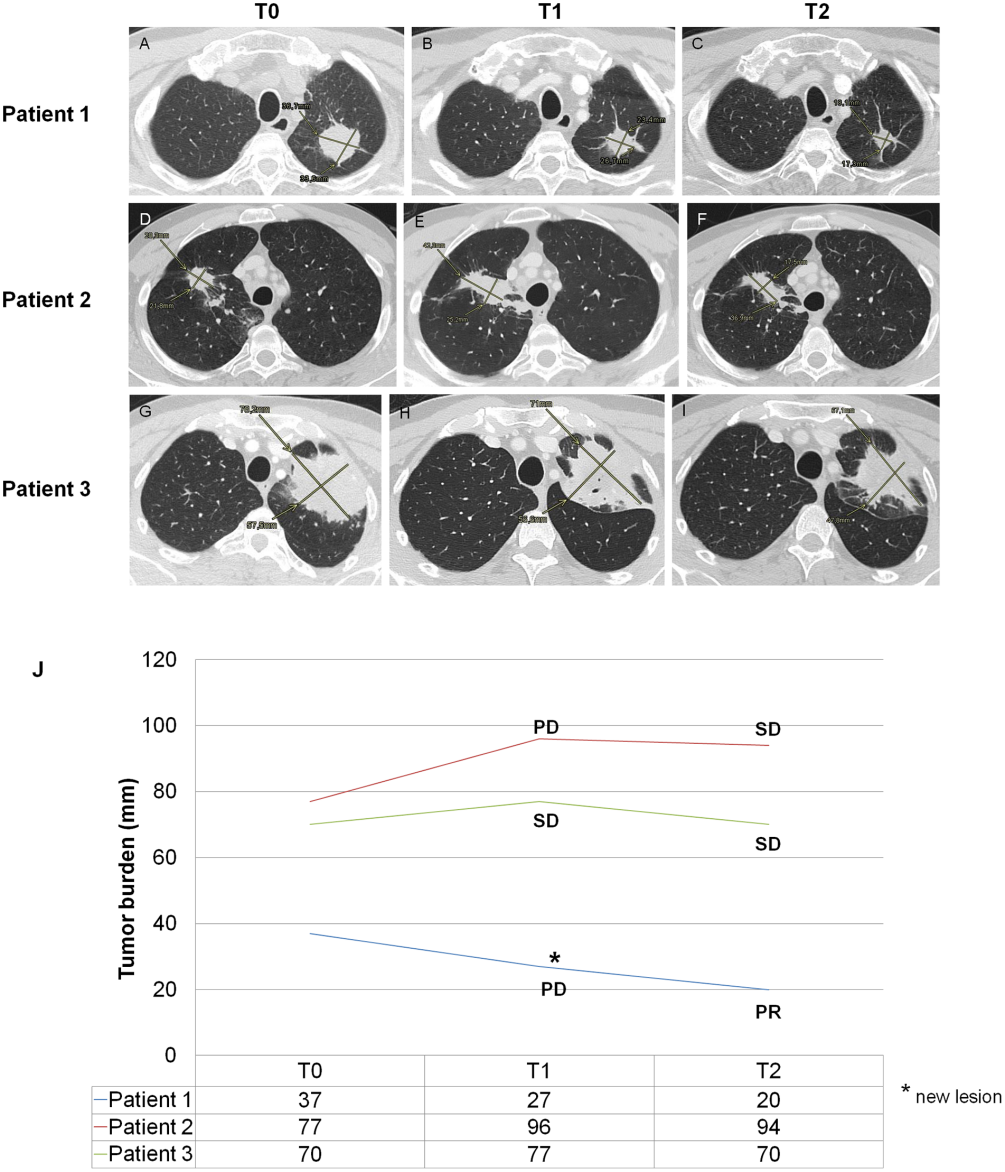
Clinical imaging. For clinical case 1, the CT scan shows a progressive size reduction of primary tumor localized in the left superior lobe **(B, C)** in comparison with baseline **(A)**. For clinical case 2, the CT scan **(E)** shows dimensional increase of primary tumor localized in the right superior lobe in comparison with baseline **(D)**. This increase could be explained as a pseudo-progression because of the reduction of the same lesion in the subsequent CT scan **(F)**. For clinical case 3, the CT scan **(H, I)** shows a size reduction of primary tumor localized in the left superior lobe in comparison with baseline **(G)**; in H, the signs of a partial cavitation (arrow) for necrosis can also be observed. **(J)** The graph shows the changes in the sum of the diameters of target lesions at T0, T1 and T2. The classification of response according to the RECIST criteria is associated with each time point. mm, millimeters; PD, progressive disease; SD, stable disease; PR, partial response.

Two months later (T1), the CT scan showed partial response on lung masses and on lymph nodes but new bone metastases were described at the spine with the onset of pain. This represents a progressive disease according with RECIST criteria. However, the patient continued the treatment beyond progression, because of the good response obtained in the thoracic lesions. Treatment-related adverse events experienced during treatment were lipase increase grade (G)2, fever and hypothyroidism G1. At the subsequent evaluation two months later (T2), he experienced further reduction of the primary lung tumor (**Figures 1A–C**, **J**).

After completing 3 cycles, the patient discontinued immunotherapy due to the confirmed radiological progression on bone and chest and a second-line treatment with platinum-based chemotherapy was started.

### Clinical Presentation of Case 2

A 54-years-old male patient was diagnosed with advanced lung adenocarcinoma in 2019. In May 2019, the CT scan showed a mass located in the right lung associated with multiple bilateral nodules and metastases in mediastinal lymph nodes (T0). The presence of these lesions was then confirmed by PET CT that also showed pleural lesions associated with costal erosive metastases.

Subsequently, the patient underwent a bronchoscopy in June 2019. Bronchial biopsies were performed and led to the diagnosis of lung adenocarcinoma. The molecular profile was negative for EGFR mutation, ALK and ROS1 rearrangements and PD-L1 expression.

A treatment with immunological triplet was started in July 2019. After two months (T1), the CT scan showed an increase of the lung lesion in the right inferior lobe, classified as progressive disease (PD) according with RECIST criteria. During the treatment, the patient experienced nausea G1 and amylase increase G1. The treatment was continued beyond progression, because of the lack of clinical worsening. At the subsequent evaluation two months later (T2), the disease was stable (**Figures 1D–F**, **J**). Subsequently, the patient underwent gamma-knife treatment for the onset of new brain metastases. The immunotherapy was interrupted until February 2020, when all pulmonary nodules were increased. A second-line treatment with platinum-based chemotherapy was started.

### Clinical Presentation of Case 3

A 57-years-old male patient was diagnosed with advanced lung adenocarcinoma. In May 2019, the CT scan showed an unresectable pulmonary mass with mediastinal lymph node involvement (T0). In June 2019, bronchial biopsies were performed and led to the diagnosis of lung adenocarcinoma. The molecular profile was negative for EGFR mutation, ALK and ROS1 rearrangements. PD-L1 expression was evaluated in two specimens, it was 70% in the first one and negative in the second one. Since July 2019, a treatment with immunological triplet was started. After two months (T1), the CT evaluation showed stable disease, even if with mild reduction of the pulmonary mass, so that the immunotherapy was continued. During this treatment, the patient experienced fever, transaminase and lipase increase G1 and subclinical hypothyroidism. At the subsequent evaluation two months later (T2), the CT scan still showed stable disease according with RECIST criteria (**Figures 1G–I**, **J**). Overall this treatment lasted for 5 cycles. In March 2020, after the finding of a reduction of pulmonary mass and remission of lymph node metastases, the patient underwent left upper lobectomy and ilo-mediastinal lymphadenectomy.

## Biological Observations

We extended our clinical evaluation to flow cytometry analysis (BD FacsCanto I, measured by FacsDiva software) of two subpopulations of MDSC-like cells (MDSC-LCs) as reported by Bronte V et al. (10) at T0, T1 and T2. In addition, we assessed the phenotypic changes of exhausted cell subsets among total circulating T, NK and NKT cells over the course of treatment. In **Supplementary Table 1**, we reported the list of antibodies, clones and conjugated fluorochromes for immunophenotypic characterization of these cell subsets.

**Figure 2:**
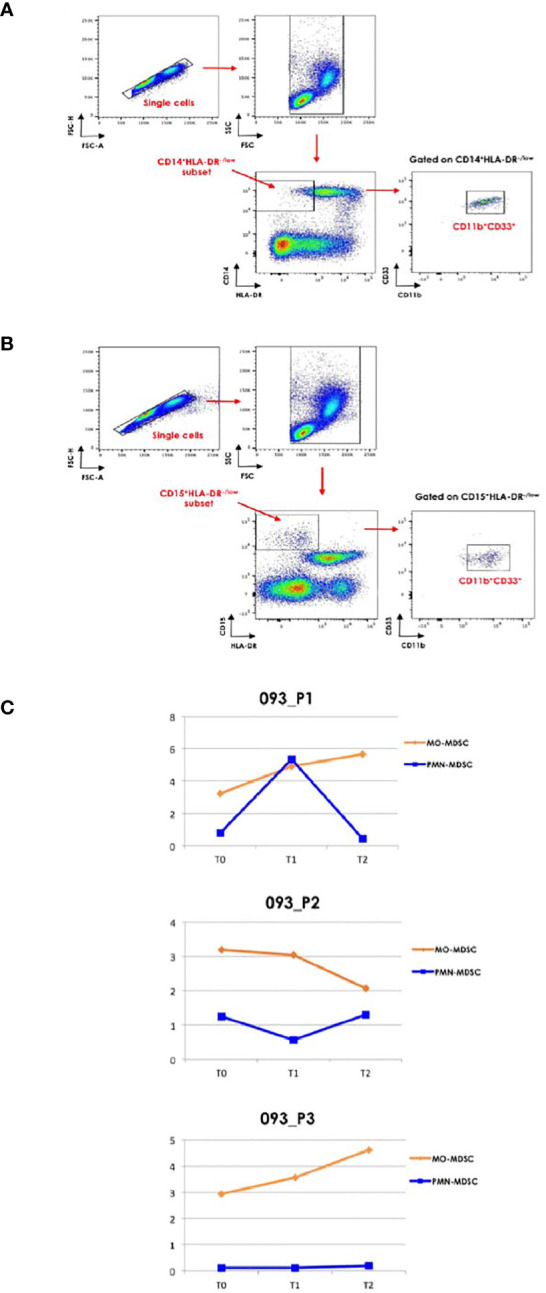
Flow cytometric characterization of MDSC. Gating strategy was used to define MDSC subpopulations. After exclusion of doublets, **(A)**, CD14^+^HLA-DR^-/low^ and **(B)**, CD15^+^HLA-DR^-/low^ cell subsets were gated and the proportion of CD11b^+^CD33^+^ was evaluated. **(C)**, The graphs report the changes in the percentage of MO-MDSC-LC and PMN-MDSC-LC in the three clinical cases at T0, T1 and T2.

**Figure 3:**
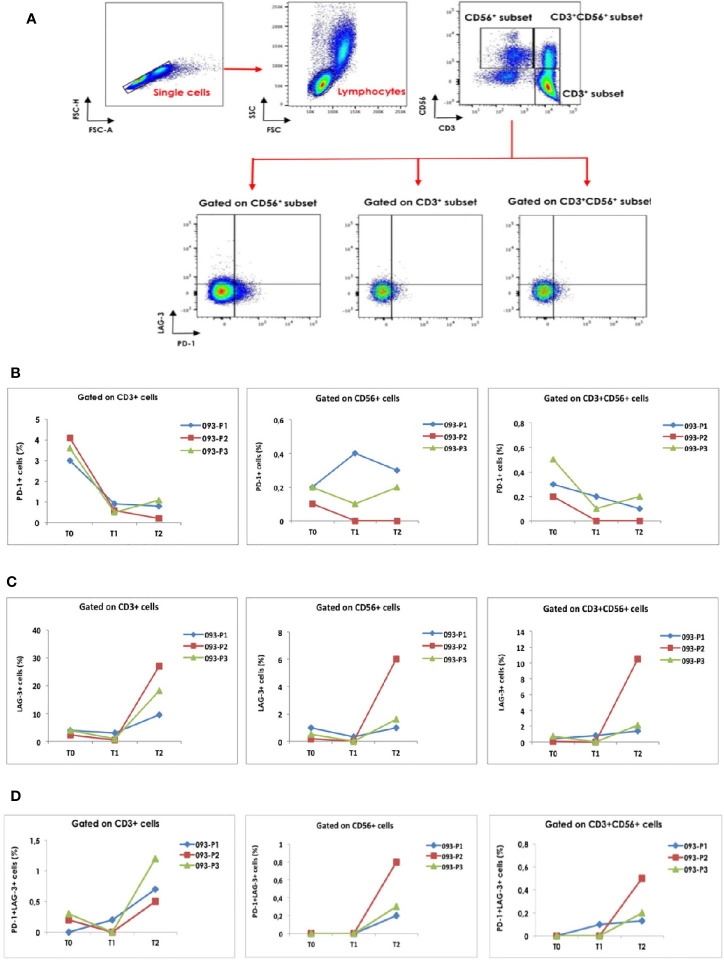
Gating strategy was used to identify **(A)**, the exhausted CD3^+^, CD56^+^ and CD3^+^CD56^+^ cell subsets by evaluating the expression of PD-1 and LAG-3. Single cells were gated and doublets were excluded. A lymphocyte gate was set in FSC-A and SSC-A plot. The graphs report the changes in **(B)**, the percentage of exhausted T, NK and NKT cells expressing PD-1, **(C)**, LAG-3 and **(D)**, both immune checkpoint molecules in the three clinical cases at T0, T1 and T2.

As reported in **Figures 2A, B**, the CD14^+^HLA-DR^-/low^ and CD15^+^HLA-DR^-/low^ cell subsets were gated and the proportion of CD11b^+^CD33^+^ was evaluated. We observed a time-dependent increase of MO-MDSC-LCs in the case 1 (093-P01) whereas the PMN-MDSC-LCs alternatively increased and decreased over the course of therapy (**Figure 2C**). In the case 2 (093-P02), the percentage of MO-MDSC-LCs reduced whereas the PMN-MDSC-LCs alternatively decreased and increased over the course of therapy (**Figure 2C**). In the case 3 (093-P03), the MO-MDSC-LCs increased whereas the PNM-MDSC-LCs remained unchanged over the course of therapy (**Figure 2C**). We also assessed the phenotypic changes of exhausted cell subsets among total circulating T, NK and NKT cells over the course of treatment. A lymphocyte gate was set based on the FSC and SSC parameters. Gating strategy was used to identify exhausted CD3^+^, CD56^+^ and CD3^+^CD56^+^ cell subsets by evaluating the expression of PD-1 or LAG-3 (**Figures 3A–C**) and both (**Figures 3A, D**). In all patients, we observed a decreased percentage of PD-1^+^ T cells whereas the percentage of LAG-3^+^ T cells increased over the course of therapy. In all patients we observed a boost of double-positive exhausted cells at T2 during the immunotherapy.

## Discussion

In this work, we analyzed the clinical features and immune evolution in three patients who received a combined treatment with ICIs for advanced NSCLC. Specifically, we evaluated the effect of ICIs treatment on the percentage of circulating MDSC-LC and exhausted T, NK and NKT cells.

The description of the three cases highlights both clinical and biological differences between the patients receiving the same schedule of treatment with ICIs.

In the clinical case 1, who experienced a partial response, we observed a boost of the percentage of PMN-MDSC-LC concomitantly with the onset of new lesions at T1, followed by their relevant decrease at T2, where we documented a further reduction of primary lung lesions. Conversely, the MO-MDSC-LC increased over the course of therapy. In the clinical case 2 who experienced a progressive disease, the clinical profile was accompanied by an unexpected behavior of MDSC-LC subpopulations. Indeed, the PMN-MDSC-LC alternatively decreased and increased, whereas the MO-MDSC-LC decreased over the course of therapy. In the clinical case 3 who experienced a stable disease, we reported low/undetectable levels of PMN-MDSC-LC and an increase of MO-MDSC-LC over the course of ICIs therapy. Various studies demonstrated that the PMN-MDSC-LCs subset was negatively associated with responsiveness to ICIs treatment (10, 11). Starting from our observation, it is unclear whether the changes in the percentage of the MDSC-LCs subsets are induced by the combination therapy.

All these observations in these 3 patients are not conclusive, but provide the evidence that the monitoring of suppressive cell subsets could help to understand the mechanisms of resistance to immune checkpoint inhibition in lung cancer patients. The combination of three different ICIs which target different molecules (i.e. CTLA-4, PD-1 and LAG-3) is a new strategy under development. Relatively to the exhausted cell subsets, we observed a discrepancy in the percentage of PD-1^+^ vs LAG-3^+^ T cells. Indeed, since both of the immune checkpoint molecules were targeted by the combination therapy, the percentage of PD-1^+^ T cells decreased over the course of therapy whereas the percentage of LAG-3^+^ T cells increased. The boost of double-positive cells may primarily result from the increased expression of LAG-3 but not PD-1 in T cells. The evaluation of these subsets should be better explored in more patients to clarify the reason that the percentage of PD-1 and LAG-3 co-expressing cells increased after the treatment independently from clinical course. In literature, a recent work conducted on 74 advanced NSCLC patients treated with nivolumab alone revealed that the frequency of exhausted T cells was higher in patients with uncontrolled disease as compared to patients with disease control. Moreover, in the group of patients experiencing disease control, the amount of exhausted T cells declined soon after the first cycle of therapy and then remained stable until the fourth administration, whereas in progressive disease group, exhausted T cell levels alternatively increased and decreased at different time points (12). However, the design of experimental models on immunotherapy could help to clarify the kinetics of these cell subpopulations with the perspective to find new targets for treatment or new biomarkers for resistance to cancer immunotherapy.

## Data Availability Statement

The original contributions presented in the study are included in the article/**Supplementary Material**. Further inquiries can be directed to the corresponding author.

## Ethics Statement

The studies involving human participants were reviewed and approved by IRST Ethics Committee (Prot. No. IRST B093; Identifier Code: L3P1752). The patients/participants provided their written informed consent to participate in this study. Written informed consent was obtained from the individual(s) for the publication of any potentially identifiable images or data included in this article.

## Author Contributions 

GB, AV, and SM: conception and design; analysis and interpretation of data; writing, review, and revision of the manuscript. AR, AA, FS, CG, MC, and MB: acquisition of data; administrative, technical, or material support; analysis and interpretation of data. AD, MM, and LC: analysis and interpretation of data; study supervision; review and revision of the manuscript. All authors contributed to the article and approved the submitted version.

## Conflict of Interest

The authors declare that the research was conducted in the absence of any commercial or financial relationships that could be construed as a potential conflict of interest.

## Supplementary Material

Supplementary Table 1 List of antibodies used for immunophenotype of MDSC-LC and exhausted immune cells, clones and conjugated fluorochromes.
