# Supplementary material for: Case Report: Circulating Myeloid-Derived Suppressive-Like Cells and Exhausted Immune Cells in Non-Small Cell Lung Cancer Patients Treated With Three Immune Checkpoint Inhibitors
Source: Front Immunol. 2021 May 27;12:672219. doi: 10.3389/fimmu.2021.672219 (PMC8191501; doi:10.3389/fimmu.2021.672219)
Supplement: Supplementary Table 1 — List of antibodies used for immunophenotype of MDSC-LC and exhausted immune cells, clones and conjugated fluorochromes. [file Table_1.docx]

| List of Antibodies | Clone | Fluorochrome |
| --- | --- | --- |
| CD14 | TÜK4 | PE |
| CD15 | VIMC6 | FITC |
| HLA-DR | REA805 | APC |
| CD11b | REA713 | PE-Vio770 |
| CD33 | REA775 | APC-Cy7 |
| CD3 | REA613 | FITC |
| CD56 | REA196 | PE |
| CD279 (PD-1) | REA1165 | PE-Vio770 |
| CD223 (LAG-3) | REA351 | APC |

**Supplementary Table 1**
